# Injury of the Knee-Joint—Inflammation—Amputation—Recovery

**Published:** 1856-10

**Authors:** H. A. Johnson

**Affiliations:** One of the Physicians to the Mercy Hospital


ARTICLE II.—*Injury of the Knee-Joint—Inflammation—Amputation—Recovery.* Treated by Dr. H. A. JOHNSON, one of the Physicians to the Mercy Hospital. Reported by P. J. Wardner.

Michael White, a native of Ireland, aged 44 years, was admitted, May 30th, with necrosis of the tibia, and severe inflammation in and about the knee-joint, which proceeded from an injury received in November last while laboring on the railroad. At the time of the injury he felt no immediate effects of a serious character, but in the month of January he fell, in passing out of the house, and gave it a severe sprain, which confined him to the bed, with much pain and swelling in the soft parts about the upper third of the tibia and outer condyle of the femur; for which he was treated by a German doctor up to the time of his admission. What his treatment had been we were unable to learn, but, when admitted, he had a severe cough, constipation of the bowels, was much reduced, and had a blister on both sides of the knee-joint. He was put upon such internal treatment as his condition required, with a view to allay the cough and regulate the bowels, after which he took tonics and alteratives, varied as circumstances required, with local applications to the part affected.

This treatment was continued up to about the 1st of July, at which time the cavity of the joint, and tissues surrounding and above it, were infiltrated with pus. The swelling began to extend up the thigh, and his general health to suffer very materially. On the 10th of July, the patient having been placed upon the operating table, Dr. Johnson made the following remarks to the students in attendance:—

"*Gentlemen*—The questions that naturally arise, are, first, as to the propriety of making any further efforts to save the limb; secondly, as to the propriety of the amputation of the femur. In reference to the first, the disease has progressed steadily and uniformly, so far as we can learn, from its commencement to the present time, notwithstanding the use of all those means which are generally followed by success in similar cases. The general condition of the patient has been such as to require constant treatment, and he is now rapidly failing. These considerations lead me to think, that it is not only useless, but that the life of the patient will be endangered by any further efforts to save the limb. While we wish to be regarded as an advocate of conservative surgery, we do not feel justified in deferring an operation when there seems to us, as in the present instance, to be a greater probability of the death of the patient without it, than the cure of the diseased member. In reference to the propriety of an operation in this case, the only question that remains to be asked, is, Does the history of the case, and the present condition of the patient, warrant us in the presumption that he will survive the operation and be ultimately restored to health? The probabilities are in his favor, therefore we shall proceed to operate."

The femur was amputated at the junction of the middle and lower third, the patient being placed under the influence of chloroform; after which he was put upon such remedies as were intended to repair the lost energies of his system. Nearly the whole extent of the cut surface united by the first intention. The ligature came away on the seventh day, and he was dismissed on the 7th of August with a perfect stump, and in good health.

